# Editorial: Metabolic dysfunction-associated steatotic liver disease (MASLD) - pathogenesis, prevention and treatment

**DOI:** 10.3389/fendo.2025.1703019

**Published:** 2025-09-24

**Authors:** Stanisław Surma, Łukasz Bułdak

**Affiliations:** Department of Internal Medicine and Clinical Pharmacology, Medical University of Silesia, Katowice, Poland

**Keywords:** MASLD, metabolic abnormalities, prevention, treatment, CV risk

Metabolic Dysfunction-Associated Steatotic Liver Disease (MASLD) represents one of the most serious health challenges facing the modern world and is currently considered the most common chronic liver disease, with a prevalence of approximately one-third of the adult population (1–3). The nomenclature change from NAFLD to MASLD was necessary to emphasize its close association with metabolic disorders such as obesity, insulin resistance, type 2 diabetes, dyslipidemia, and hypertension, which not only increase the risk of developing the disease but also determine its progression and complications (1). MASLD is an insidious disease, often developing asymptomatically, and its diagnosis is often made only in advanced stages, when metabolic dysfunction-associated steatohepatitis (MASH), fibrosis, cirrhosis, or hepatocellular carcinoma develop. Furthermore, MASLD significantly increases the risk of cardiovascular and renal diseases, which in clinical practice are the leading causes of death in this group of patients (2, 3). The global burden of MASLD continues to grow, generating significant health and economic consequences, including the costs of treating complications, decreased productivity, and premature death (2, 3). In this context, an interdisciplinary approach encompassing basic research, clinical diagnostics, pharmacotherapy development, and public health interventions is crucial. This Research Topic, titled “Metabolic Dysfunction-Associated Steatotic Liver Disease (MASLD) – Pathogenesis, Prevention, and Treatment,” comprises thirteen studies that collectively provide a multidimensional picture of the disease, highlighting pathogenic mechanisms, risk factors, diagnostic tools, therapeutic options, social determinants, and implications for healthcare systems.

Several studies have made significant contributions in the area of ​​pathogenesis and risk factors. Shen et al. conducted a large population-based study that demonstrated that elevated aldosterone levels are associated with a higher prevalence of MASLD in patients with arterial hypertension. This finding highlights the role of the endocrine system in the development and progression of the disease and suggests the possibility of incorporating parameters related to mineralocorticoid metabolism into future risk assessment algorithms. Chen et al. focused on the atherogenic index of plasma (AIP), which reflects the balance between lipid fractions in serum. Analysis of NHANES data showed that a high AIP is associated with an increased incidence of MASLD, further supporting the importance of lipid abnormalities in the development of this condition. Meroni et al. in a literature review of patients with MASLD and hepatocellular carcinoma, presented evidence indicating that the presence of cardiometabolic factors significantly accelerates neoplastic progression and worsens prognosis, highlighting the urgent need for comprehensive monitoring of patients with MASLD for oncological risk. Diagnosing MASLD remains a challenge, especially since the disease develops insidiously and affects a large population. Hong et al. demonstrated that Golgi protein 73 (GP73) may be a useful marker of liver fibrosis, capable of supporting the assessment of disease severity without the need for biopsy. Alkaabi et al. assessed the effectiveness of simple predictive tools, such as Fib-4 and the Hamaguchi score, in identifying MASLD in patients with type 2 diabetes, which has important implications for clinical practice in diabetes, enabling rapid and accessible methods for early detection of the disease. Sharma et al. used FibroScan elastography among medical students and correlated its results with 30-year Framingham risk scores, demonstrating that MASLD can be considered an early indicator of overall cardiometabolic risk even in young populations. This creates potential opportunities for early screening of young people and implementation of interventions aimed at reducing the risk of premature heart attack (4).

In terms of therapy and prevention, this Research Topic provides inspiring data. Suzuki et al. demonstrated that the use of pemafibrate in patients with type 2 diabetes led to improvement in both the fatty liver index and lipid profile, indicating that this drug may play an important role in the treatment of MASLD. Oe et al. described the case of a patient with severe MASLD, in whom switching from standard GLP-1 agonist therapy to tirzepatide led to significant clinical and biochemical improvement. This paper provides proof of concept for the potential efficacy of new dual GIP/GLP-1 agonists in the treatment of advanced MASLD. Taiwo et al. addressed the issue of MASLD in the context of type 1 diabetes, demonstrating characteristic metabolomic abnormalities. This is an important finding, as most studies have focused on type 2 diabetes, yet patients with T1DM also constitute a risk group requiring a tailored diagnostic and therapeutic approach. Hu et al. focused on children and adolescents, demonstrating that the distribution of body fat, not just overall BMI, determines the risk of MASLD. These results indicate the need to implement targeted preventive measures already in childhood, which could limit the progression of the disease in adulthood. Social and environmental factors are another area that cannot be ignored. Wang et al. using NHANES data from 1999–2014, demonstrated that long work hours and the specific nature of employment significantly increase the risk of MASLD. These results highlight the need for public health interventions that also take into account working conditions and the lifestyle of the population. Zhu et al. demonstrated that low folate and vitamin B12 levels are associated with increased mortality in patients with MASLD, suggesting that supplementation and monitoring vitamin status may be simple and effective interventions to improve prognosis. Anastasaki et al. developed and tested an algorithm for primary care in a European setting that allows for early diagnosis and more effective management of MASLD. Implementation of such solutions could significantly improve the quality of care for patients in the general population.

In summary, the articles collected in this Research Topic demonstrate MASLD as a disease with a complex, systemic pathogenesis and a wide spectrum of complications, requiring a multifaceted approach (**Table 1**). On the one hand, the development of biomarkers and non-invasive diagnostic methods allows for earlier detection of the disease, while advances in pharmacotherapy and personalized treatment offer new therapeutic options. The importance of social and environmental factors, which play a key role in shaping the risk of MASLD and should be considered in preventive measures, cannot be overlooked. Future research on MASLD should focus on integrating clinical, molecular, and epidemiological data to create personalized treatment and prevention strategies. Developing screening programs for high-risk populations and health education across various age and social groups is also essential. This collection of thirteen articles represents a significant step forward in MASLD research, providing scientific evidence that can translate into clinical practice and health policy. We believe that these results will contribute to improving the diagnosis, treatment and prevention of MASLD and, consequently, to reducing the global burden of this rapidly growing epidemic.

**Table 1:** Summary of articles published in Research Topic.

Author; year; ref	Topic	Key findings	Implication
Shen et al.; 2024	Aldosterone and MASLD	High aldosterone levels increase the incidence of MASLD in patients with HT	Possibility of inclusion in risk assessment
Chen et al.; 2024	AIP and MASLD	High AIP associated with MASLD in the NHANES population	Atherosclerosis prediction using AIP in MASLD patients
Meroni et al.; 2024	MASLD and HCC	Cardiometabolic factors accelerate cancer progression	The need to monitor the oncological risk of patients with MASLD
Hong et al.; 2025	GP73 and fibrosis in MASLD	A new biomarker of fibrosis in MASLD	Enrichment of non-invasive diagnostic methods
Alkaabi et al.; 2024	Predictive tools	Fib-4 and Hamaguchi effective in T2DM	Application in primary care and diabetology
Sharma et al.; 2024	FibroScan and Framingham	MASLD as an indicator of cardiometabolic risk in young adults	Possibility of screening and early optimization of cardiovascular risk
Suzuki et al.; 2025	Pemafibrate in T2DM patients	Improvement of steatosis index and lipid profile in T2DM	New pharmacotherapy option
Oe et al.; 2025	Tirzepatide (case report)	Improving the outcomes of patients with severe MASH	Proof of concept for GIP/GLP-1 efficacy; new pharmacotherapy option
Taiwo et al.; 2025	Metabolomics in T1DM patients with MASLD	Specific metabolic disorders	Personalization of MASLD diagnostics in patients with T1DM
Hi et al.; 2025	Children’s body structure	Segmental adipose tissue as a predictor of MASLD	Early pediatric prevention
Wang et al.; 2025	Working time	Long hours and type of work increase the risk of MASLD	Considering occupational factors in MASLD risk assessment
Zhu et al.; 2024	Folate and vitamin B12 and mortality in MASLD	Deficiencies increase mortality in MASLD	Simple supplementation intervention
Anastasaki et al.; 2024	Algorithm for the diagnosis and management of MASLD in primary care practice	The use of algorithms helps in the management of MASLD	Improving diagnosis and care

## References

[1] Dale K Fallouh Y Alkhouri N. MASLD and MASH: how a change of nomenclature may impact our approach in treating liver disease. Expert Opin Investig Drugs. (2024) 33:1095–7. doi: 10.1080/13543784.2024.2401907, PMID: 39256989

[2] Younossi ZM Kalligeros M Henry L. Epidemiology of metabolic dysfunction-associated steatotic liver disease. Clin Mol Hepatol. (2025) 31:S32–50. doi: 10.3350/cmh.2024.0431, PMID: 39159948 PMC11925440

[3] Bołdys A Bułdak Ł Maligłówka M Surma S Okopień B. Potential therapeutic strategies in the treatment of metabolic-associated fatty liver disease. Med (Kaunas). (2023) 59:1789. doi: 10.3390/medicina59101789, PMID: 37893507 PMC10608225

[4] Banach M Surma S. Monitoring of traditional atherosclerosis cardiovascular disease risk factors - is it enough to prevent premature acute coronary syndrome? Lancet Reg Health Eur. (2024) 38:100866. doi: 10.1016/j.lanepe.2024.100866, PMID: 38476739 PMC10928265

